# The Hippo pathway and cancer immunity: friend or foe?

**DOI:** 10.18632/oncoscience.398

**Published:** 2018-04-29

**Authors:** Helena J Janse van Rensburg, Xiaolong Yang

**Affiliations:** Department of Pathology and Molecular Medicine, Queen's University, Kingston, Ontario, Canada K7L 3N6

**Keywords:** Hippo pathway, TAZ, YAP, PD-L1, immune evasion

Within the past century, the roles of the immune system in cancer development and progression have become increasingly clear [1]. It is now well-appreciated that cancer cells and immune cells interact within the tumour microenvironment as immune cells impose a selective pressure on cancer cells and, in turn, cancer cells modulate immune cell functions to escape destruction. A greater understanding of the molecular mechanisms underlying these interactions has led to the development of novel immunotherapies that have generated tremendous excitement in the field of cancer research. Despite this, questions remain about how key targets of immunotherapies such as the immune checkpoint molecule PD-L1 are regulated in the context of cancer. Furthermore, whether the regulatory mechanisms for these potential biomarkers are conserved between human cancers and animal models of cancer is unclear.

The Hippo signaling pathway has recently emerged as a pivotal player in cancer biology. In this pathway, MST1/2 kinases phosphorylate and activate a second set of kinases, LATS1/2. LATS1/2 subsequently phosphorylate two transcriptional co-activators, TAZ and YAP, leading to their sequestration in the cytoplasm, degradation and functional inhibition. The Hippo pathway effectors, TAZ and YAP, are oncogenes that are commonly dysregulated in cancer [2]. We and others have previously found that TAZ/YAP regulate the expression of many gene targets that are involved in processes like cell proliferation, cell migration, evasion of apoptosis, cancer stem cell phenotypes and chemotherapy resistance. Therefore, TAZ and YAP have evident cancer cell-intrinsic functions in tumourigenesis. However, relatively less is known about how dysregulated TAZ or YAP expression can affect heterotypic interactions of cancer cells with stromal cells in the tumour microenvironment. Indeed, few studies have explored how Hippo signaling alters the relationship between cancer cells and tumour-infiltrating immune cells. In our recent study [3], we performed a screen for immune-related gene targets of TAZ and YAP in breast cancer cells using NanoString gene expression profiling. We found that TAZ or YAP overexpression in MCF10A induced dramatic changes in the mRNA expression of many genes that are relevant to immunology including *S1PR1*, *PDGFB*, *NLRP3* and the immune checkpoint molecules *PD-L1* and *PD-L2*. We further validated *PD-L1* as a *bona fide* transcriptional target of TAZ in human breast cancer cells. Overexpression of TAZ (or knockdown of MST1/2 or LATS1/2) increased PD-L1 expression in TAZ-low/PD-L1-low cell lines whereas loss of TAZ (or LATS1/2 overexpression) reduced PD-L1 in TAZ-high/ PD-L1-high cell lines. Mechanistically, we showed that TAZ physically interacts with the *PD-L1* promoter through the TEAD family of transcription factors and enhances its activity. Notably, TAZ-induced PD-L1 expression had functional significance in co-culture experiments where cancer cell TAZ expression enhanced T cell apoptosis and suppressed T cell IL-2 production.

Our observation that TAZ can promote human cancer immune evasion through PD-L1 is supported by several recent publications [4, 5, 6, 7] but lies in stark contrast to work performed by Moroishi *et al.* [8]. In their report, Moroishi *et al*. showed that loss of LATS1/2 (or overexpression of TAZ or YAP) in fact enhances the anti- cancer immune response in three mouse syngeneic tumour models. The authors demonstrated that LATS1/2 genetic knockout causes murine cancer cells to release nucleic acid rich extracellular vesicles that can stimulate dendritic cells and improve antigen cross-presentation to T cells. While a definitive explanation reconciling these findings with our own has not been established, it is possible that the choice of model systems in our respective studies may be a factor. Given that we were unable to observe the relationship between TAZ and PD-L1 in mouse cell lines, we suspect that there may be broad species-specific differences in gene regulation by the Hippo pathway. Consistent with this notion, we found very little overlap in TAZ-regulated immune-related genes when we performed a second NanoString screen in two mouse mammary cell lines. These findings point to a model in which the Hippo pathway regulates different gene targets across species. While enticing, the potential for species-specific gene targets of the Hippo pathway and, perhaps, species- specific functions for the Hippo pathway has yet to be explored in the literature. More comprehensive efforts to compare Hippo pathway targets across species using multiple cell lines and model systems will certainly be warranted.

New roles for the Hippo pathway in modulating immune responses continue to emerge. In our own work, we identified a relationship between TAZ and PD-L1 expression in human cancer cell lines and implicated the Hippo pathway effector TAZ in human cancer immune evasion. Future work will be necessary in order to situate this finding within the existing literature so that we can develop a complete understanding of how this pathway functions in cancer biology.
